# Inverse associations of the lifestyle critical 9 with cardiorenal syndrome: the mediating role of the dietary inflammatory index

**DOI:** 10.3389/fnut.2025.1519612

**Published:** 2025-03-13

**Authors:** Hongman Li, Long Li

**Affiliations:** ^1^Department of Rheumatology and Immunology, The Affiliated Hospital of Guizhou Medical University, Guiyang, China; ^2^Clinical Medical College, Guizhou Medical University, Guiyang, China

**Keywords:** cardiorenal syndrome, Life’s Crucial 9, dietary inflammatory index, mediation analysis, NHANES

## Abstract

**Background:**

Cardiorenal syndrome (CRS) represents a burgeoning global health concern characterized by its increasing prevalence. Life’s Crucial 9 (LC9), an innovative tool for cardiovascular health assessment, and the Dietary Inflammatory Index (DII), which quantifies diet’s impact on body inflammation, have not been previously studied in conjunction regarding their association with CRS.

**Objective:**

This study aims to explore the relationship between LC9 and CRS, using data from the National Health and Nutrition Examination Survey (NHANES), and to examine whether DII serves as a mediator in this association.

**Methods:**

This research included data from 25,792 NHANES participants spanning from 2005 to 2018. The study leverages the dataset’s comprehensive representativeness and robust statistical power to ensure generalizable and reliable findings. We employed weighted logistic regression to evaluate the association between LC9 scores and CRS presence, conducted subgroup analyses, and performed mediation analysis to investigate the role of DII.

**Results:**

Our analysis demonstrated a significant inverse relationship between LC9 and CRS. Upon controlling for confounders, each 10-point rise in LC9 correlates with a 26% reduction in CRS prevalence (*p* < 0.001). Additionally, stratifying LC9 into tertiles with T1 as the reference group revealed that T2 (OR = 0.59, 95% CI = 0.48–0.72, *p* < 0.001) and T3 (OR = 0.57, 95% CI = 0.38–0.88, *p* < 0.001) exhibited a strong negative correlation trend. The dose–response curve illustrates a linear relationship between LC9 and CRS; as LC9 increases, the occurrence of CRS decreases. DII shows a significant positive connection with CRS (*p* < 0.001), but DII indicates a decreasing trend when LC9 rises (*β* = −0.65, *p* < 0.001). Mediation analysis reveals that DII mediates the association between LC9 and CRS, with a mediation proportion of 12.5% (*p* < 0.001).

**Conclusion:**

The findings indicate a robust inverse correlation between LC9 scores and CRS incidence, with DII is associated with this relationship. This suggests potential preventive strategies against CRS through lifestyle modifications guided by LC9.

## 1 Introduction

Cardiorenal syndrome (CRS) refers to a clinical syndrome caused by impaired interactions between the heart and kidneys (1). As the population ages and the burden of chronic diseases grows, the incidence and clinical impact of CRS are also increasing. CRS adversely impacts patients’ quality of life and substantially escalates the utilization of medical resources and associated healthcare costs. According to the 2009 Acute Dialysis Quality Initiative, it is divided into five subtypes (2), involving the interactions between acute and chronic heart and kidney conditions. Type 1 and 2 CRS arise from cardiogenic factors affecting renal function, whereas Type 3 and 4 are due to nephrogenic factors impacting cardiac function. Type 5 CRS includes cardiac and renal dysfunctions caused by systemic diseases. The global prevalence of CRS has increased (3), especially among heart failure patients, where the prevalence exceeds 50% (4). Cardiovascular-related deaths account for nearly half of all deaths among individuals with chronic kidney disease (CKD) (5). Furthermore, the simultaneous occurrence of heart failure and kidney disease heightens this risk (4, 6, 7). Between 2011 and 2020, the United States saw 97,135 deaths related to cardiorenal diseases in adults aged 15 and older (8). This presents considerable management difficulties for public health and healthcare systems. Current research has revealed the complexity of the pathophysiological mechanisms of CRS, involving various factors such as neuroendocrine activation, inflammatory pathways, oxidative stress, and biomechanical changes (9). These mechanisms are not only interconnected with each other but also interact with multi-organ dysfunction, leading to a vicious cycle of disease. To reduce the global disease burden and improve public health levels, identifying the potential risk factors of CRS through epidemiological research is crucial.

In 2010, the American Heart Association (AHA) introduced the public health initiative Life’s Simple 7 (LS7), designed to enhance cardiovascular health (CVH) by addressing seven determinants: smoking, weight, total cholesterol, blood glucose, physical activity, and food (10). Life’s Essential 8 (LE8) is a revised and more extensive health evaluation framework introduced by the AHA in 2022. This model incorporates the essential element of “sleep” into the prior LS7, highlighting the significance of enough and high-quality sleep for CVH (11). Current depression is a prevalent and serious mental health disorder, impacting approximately 300 million people globally. The World Health Organization predicts that by 2030, major depressive disorder will become the leading factor in the global burden of chronic diseases (12). Therefore, many studies emphasize the importance of depression in the prevention of cardiovascular disease (CVD) (13, 14) and introduce another new indicator, Life’s Crucial 9 (LC9), which integrates mental health into LE8 (15). The score range for these nine components is 0 to 100, and the total score for LC9 is determined as the arithmetic mean of the 9 indications.

Currently, there is no research on LC9 and CRS, but previous evidence suggests that higher LE8 levels are associated with a lower likelihood of CVD, CKD, and their related mortality rates (16–19). Additionally, research has established a correlation between depression and the incidence of CVD and CKD (13, 20). Alexander A. Huang and colleagues used data from the NHANES database for the years 2017–2020 to identify risk factors for coronary artery disease using the powerful machine learning model XGBoost. Out of a total of 684 features, 58 were found to be significant in univariate analysis (*p* < 0.0001), including components of LC9 such as blood pressure, blood lipids, blood sugar, smoking, obesity, depression, and diet (21). Therefore, we speculate that the LC9 guidelines are closely related to the prevention and management of CRS. By adhering to the LC9 guidelines, the risk of CRS can be effectively reduced, thereby improving the quality of life for patients.

Systemic inflammation is widely recognized as a critical factor in the onset and progression of CRS (9). Additionally, more and more studies are currently confirming that diet significantly influences the regulation of systemic inflammation (22). Dietary anti-inflammatory components such as omega-3 fatty acids (primarily found in deep-sea fish), high-quality dietary fibre, antioxidants (such as vitamin E, vitamin C, and beta-carotene), and polyphenols (abundant in fruits, vegetables, nuts, tea, and coffee) can reduce the levels of inflammation markers in the body. These nutrients work by reducing oxidative stress and regulating immune function to suppress inflammation (23). Additionally, consuming a diet rich in monounsaturated and polyunsaturated fatty acids, high in potassium, and low in sodium is associated with improvements in blood lipids, blood pressure, and blood sugar levels, thereby being related to a reduced risk of cardiovascular diseases and chronic kidney disease (24). The Dietary Inflammatory Index (DII) is a scientific metric introduced in 2014, derived from an extensive study of scientific literature, to evaluate the influence of diet on inflammatory status. It evaluates various food components according to their enhancing or suppressing effects on inflammatory markers (IL-1β, IL-4, IL-6, IL-10, TNF-*α*, and C-reactive protein) (25). A higher DII score signifies a diet with pronounced pro-inflammatory characteristics, whereas a lower or negative score denotes anti-inflammatory features in the diet. Since its inception, the DII has been extensively utilized in epidemiological and clinical research to investigate the correlation between dietary inflammatory potential and diverse health outcomes (26, 27). A healthy diet occupies an important position in LC9, emphasizing the intake of foods rich in fruits, vegetables, whole grains, low-fat proteins, and healthy fats. This dietary approach is usually associated with a low DII score. Nutrition significantly influences CVD and CKD. Unhealthy dietary patterns (most notably the high-fat, high-calorie Western diet) can exacerbate chronic low-grade inflammation, thereby promoting abnormal immune activation and contributing to the onset of CVD (28). A meta-analysis indicates that a 1-point increase in the DII score is related to an 8% rise in the risk and mortality linked to CVD, highlighting the substantial influence of an anti-inflammatory diet on CVD prevention (29). The inflammation associated with CKD has also been confirmed to be related to DII (30). At present, there is an absence of extensive epidemiological research capable of thoroughly investigating the pathophysiology of CRS to inform preventative and therapy efforts (31). Consequently, we undertook this study, employing a large cross-sectional methodology for the first time to examine the relationship between LC9 and the occurrence of CRS, while also investigating whether this relationship is mediated by DII, with the objective of determining whether enhancing LC9 is associated with a reduced occurrence of CRS.

## 2 Methods

### 2.1 Study population

**Figure 1:**
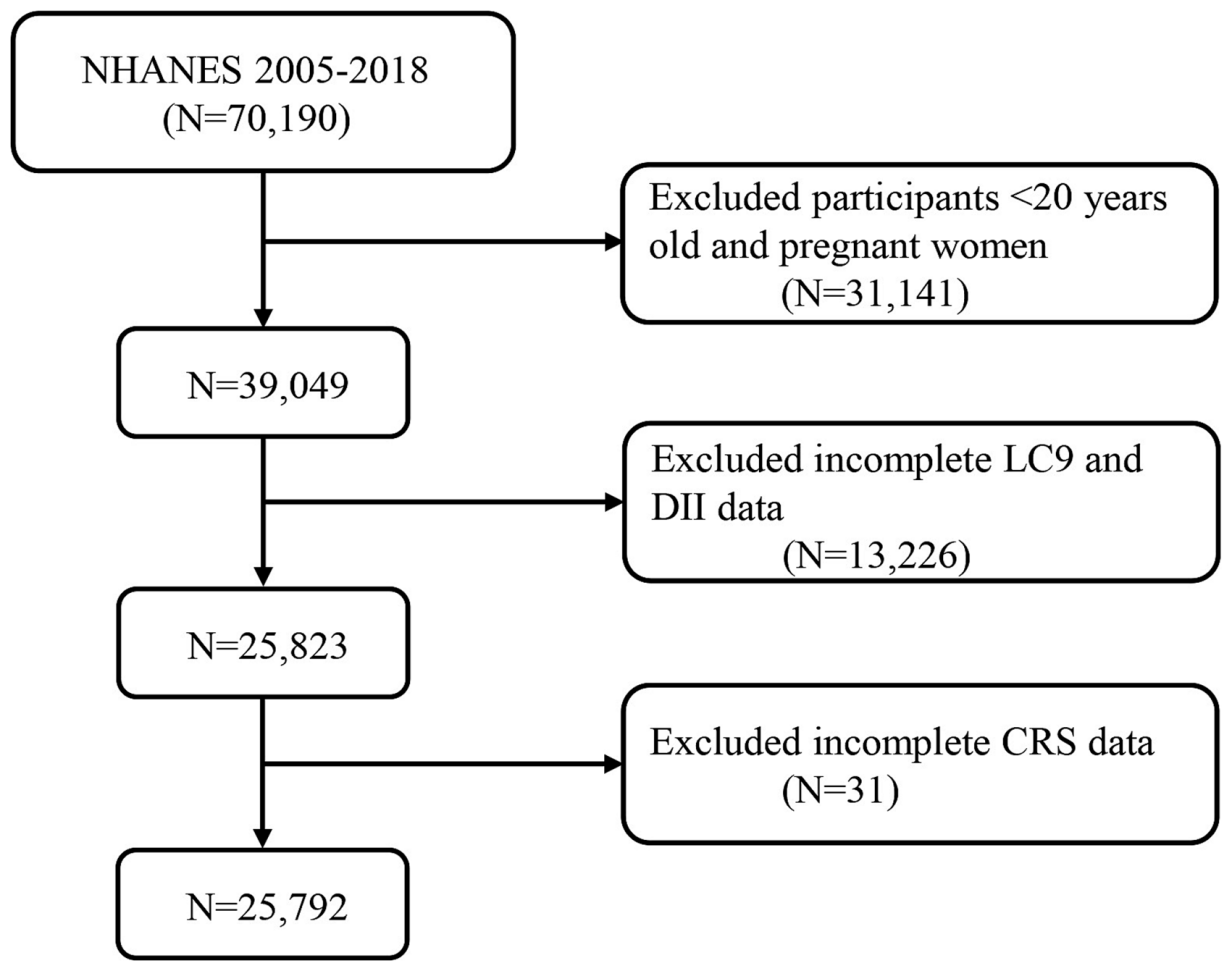
A flow diagram of eligible participant selection in the national health and nutrition examination survey. LC9, life’s crucial 9; DII, dietary inflammatory index; CRS, cardiorenal syndrome.

The cross-sectional data comes from the National Health and Nutrition Examination Survey (NHANES), a series of research projects managed by the National Center for Health Statistics (NCHS). This survey intends to evaluate the health and nutritional status of the U.S. population by collecting health-related data to help formulate public health policies, guide disease prevention programs, and improve health services. This research employed data from seven cycles of the NHANES, covering the years 2005 to 2018, with a total of 70,190 participants. Individuals under 20 are still developing, with differences in metabolism, dietary habits, and physical activity compared to adults. Using 20 years as a cutoff helps focus on chronic disease risk factors (32). Pregnant women have distinct nutritional needs and metabolic changes, which affect their diet and health outcomes, and medications or supplements taken during pregnancy can confound results (33). This classification is commonly used in public health studies, allowing for easier comparison with other research (34). As a result, we excluded participants under 20, pregnant women (31,141), those missing LC9 and DII data (13,226), and those with missing CRS data, resulting in 25,792 participants for the final analysis (Figure 1). NHANES received approval from NCHS, and all subjects gave their informed consent. The information utilized for this investigation can be found at (https://www.cdc.gov/nchs/nhanes/index.htm).

### 2.2 Evaluation criteria for LC9

LC9 adds depression as an evaluation metric based on the LE8 score. LE8 consists of 4 health behaviors (diet, physical activity, nicotine exposure, and sleep duration) and 4 health factors (body mass index, non-high-density lipoprotein cholesterol, blood glucose, and blood pressure). The calculation method for the LE8 score of each indicator has been detailed in previous publications (35). In short, each of the 8 CVH indicators is allocated a score from 0 to 100, which is determined by expert panel members using a modified Delphi method based on health outcomes and risk associations. The overall LE8 score is the average of the 8 indicators. The increased depression score of LC9 is calculated based on the Patient Health Questionnaire-9 (PHQ-9) score, which is a validated structured questionnaire used for depression screening. A higher PHQ-9 score indicates the presence of a higher level of depressive symptoms. The depression scores are categorized as 100, 75, 50, 25, and 0, corresponding to PHQ-9 scores of 0–4, 5–9, 10–14, 15–19, and 20–27, respectively. The total LC9 score is calculated as the arithmetic mean of the 9 indicators (36). Detailed classification and scoring methods can be found in Supplementary Tables 1, 2.

### 2.3 Evaluation criteria of DII

The Mobile Examination Center recorded the types and quantities of food and beverages consumed by participants in the 24 h preceding the interview, which were used to calculate the DII (27). In this study, 28 out of 45 food parameters were included: alcohol, *β*-carotene, caffeine, carbohydrate, cholesterol, energy, total fat, fiber, folic acid, iron, magnesium, monounsaturated fatty acids, polyunsaturated fatty acids, n-3 fatty acids, n-6 fatty acids, protein, saturated fat, selenium, zinc, vitamin A, B1, B2, B3, B6, B12, C, D, and E (37). Shivappa and his colleagues indicated that utilizing no more than 30 dietary characteristics remains adequate for maintaining the prediction validity of the DII for diet-related inflammation (38). The DII calculation relies on dietary intake statistics, which are correlated with a geographically representative global database that offers a reliable estimate of the mean and standard deviation for each parameter (Supplementary material).

### 2.4 Assessment of CRS

Based on prior research, we define CRS as people concurrently diagnosed with both CVD and CKD (39). CVD is identified through self-reported diagnoses of coronary heart disease, angina, congestive heart failure, myocardial infarction, or cerebrovascular accident. Chronic Kidney Disease (CKD) is defined by an estimated glomerular filtration rate (eGFR) of less than 60 mL/min/1.73 m^2^. We employed the CKD-Epidemiology Collaboration (EPI) equation to compute eGFR (40).

### 2.5 Covariables

We included age, gender, race, married/live with partner, education level, poverty income ratio, smoking, drinking, hypertension, diabetes, and hyperlipidemia as covariates. Specific classifications can be found in Supplementary Table 3.

### 2.6 Statistical analyses

All data in this study were subjected to statistical analysis utilizing R software (version 4.3.1). We utilized weights for guaranteeing that our survey information is nationally representative. In descriptive statistical analysis, participants were classified into non-CRS and CRS groups, using mean ± standard deviation (SD) to describe continuous variables and applying the student’s t-test to evaluate differences between the two groups. For categorical variables, we utilize frequency and percentage descriptions, employing the chi-square test for comparison analysis.

We utilized a weighted multivariable logistic regression model to investigate the relationship between LC9 and CRS, with LC9 being the tertile. Further calculate the *p*-values for the trend test and linear trend to determine their relationship. This research established: Model 1, the unadjusted original model. Model 2 adjusted for age, gender, education level, marital status, and race. Model 3 further adjusted for income, smoking, drinking, hypertension, diabetes, and hyperlipidemia. We stratified LC9 and DII into tertiles, employing the initial tertile as the reference group for the trend analysis. A smooth curve fitting method has been used for analyzing the possible linear correlation of LC9 and CRS. Furthermore, odds ratios (ORs) were computed with each 10-point increment in LC9, and analyses of subgroups were performed. To verify the robustness of the results, this study performed sensitivity analysis using multiple imputation with chained equations (MICE). The main analysis was repeated using five imputed datasets to address missing data for LC9, DII, and CRS.

The R software’s “mediation” package is employed to assess indirect, direct, and total effects. Mediation analysis with 1,000 bootstrap resamples was performed, adjusting variables to explore if DII acts as a mediator in the relationship between LC9 and CRS. The proportion mediated is calculated using the equation: (indirect effect / (indirect effect + direct effect)) × 100% (41). The overall effect of LC9 on CRS (Path C), LC9’s direct effect on CRS when DII is included as a mediator (Path C′), the impact of LC9 on DII (Path A), the influence of DII on CRS (Path B), and the indirect influence of DII on the LC9-CRS relationship (Path A*B) are all expressed through regression coefficients.

## 3 Results

### 3.1 Characteristics of the participants

A total of 25,792 participants satisfied the criteria for this cross-sectional study, with data appropriately weighted. Among these, 96% did not have CRS, while 4% were diagnosed with CRS. The prevalence of persons with CRS markedly escalated with age. A greater prevalence of CRS was noted in males, non-Hispanic White people, persons who were unmarried or not cohabiting, those living in poverty, smokers, and individuals with hypertension, diabetes, or hyperlipidemia (*p* < 0.05). Persons with CRS exhibited reduced LC9 scores (*p* < 0.05) and elevated DII scores (*p* < 0.05) (Table 1).

**Table 1:** Baseline characteristics of all participants were stratified by CRS, weighted.

Characteristic	Overall, *N* = 121,376,247 (100%)	Non-CRS, *N* = 117,046,537 (96%)	CRS, *N* = 4,329,710 (4%)	*p* value
**Age (%)**				**<0.001**
20–40	42,456,712 (35%)	42,413,276 (36%)	43,436 (1.0%)	
41–60	45,778,352 (38%)	45,119,422 (39%)	658,930 (15%)	
>60	33,141,183 (27%)	29,513,840 (25%)	3,627,344 (84%)	
**Gender (%)**				**0.034**
Male	58,880,653 (49%)	56,622,619 (48%)	2,258,035 (52%)	
Female	62,495,594 (51%)	60,423,919 (52%)	2,071,675 (48%)	
**Race (%)**				**<0.001**
Non-Hispanic White	86,079,973 (71%)	82,649,099 (71%)	3,430,874 (79%)	
Non-Hispanic Black	12,404,256 (10%)	11,908,826 (10%)	495,429 (11%)	
Other	13,977,005 (12%)	13,716,139 (12%)	260,866 (6%)	
Mexican American	8,915,013 (7%)	8,772,473 (7%)	142,541 (4%)	
**Married/live with partner (%)**				**<0.001**
No	43,335,340 (36%)	41,398,334 (35%)	1,937,007 (45%)	
Yes	78,040,907 (64%)	75,648,204 (65%)	2,392,703 (55%)	
**Education level (%)**				**<0.001**
Below high school	16,944,388 (14%)	15,822,817 (14%)	1,121,571 (26%)	
High school or above	104,431,860 (86%)	101,223,720 (86%)	3,208,139 (74%)	
**PIR (%)**				**<0.001**
Not poor	91,911,872 (81%)	88,871,673 (81%)	3,040,200 (75%)	
poor	22,085,636 (19%)	21,085,746 (19%)	999,890 (25%)	
**Smoking (%)**				**<0.001**
Never	66,269,886 (55%)	64,532,533 (55%)	1,737,354 (40%)	
Former	31,912,778 (26%)	30,016,697 (26%)	1,896,082 (44%)	
Current	23,193,583 (19%)	22,497,309 (19%)	696,274 (16%)	
**Drinking (%)**				**<0.001**
Former	16,149,048 (14%)	14,847,761 (13%)	1,301,287 (32%)	
Heavy	24,004,752 (20%)	23,708,170 (21%)	296,581 (7.3%)	
Mild	44,787,258 (38%)	43,188,644 (38%)	1,598,614 (39%)	
Moderate	20,896,804 (18%)	20,590,710 (18%)	306,093 (7.5%)	
Never	12,294,316 (10%)	11,724,219 (10%)	570,097 (14%)	
**Hypertension (%)**				**<0.001**
No	74,048,262 (61%)	73,485,482 (63%)	562,780 (13%)	
Yes	47,327,986 (39%)	43,561,056 (37%)	3,766,930 (87%)	
**Diabetes (%)**				**<0.001**
No	105,950,276 (87%)	103,686,072 (89%)	2,264,204 (52%)	
Yes	15,425,972 (13%)	13,360,466 (11%)	2,065,506 (48%)	
**Hyperlipidemia (%)**				**<0.001**
No	34,387,347 (28%)	34,050,300 (29%)	337,048 (7.8%)	
Yes	86,988,900 (72%)	82,996,238 (71%)	3,992,662 (92%)	
**LC9 [mean (SD)]**	70.42 (13.62)	70.84 (13.44)	59.03 (13.68)	**<0.001**
**LC9, tertile (%)**				**<0.001**
T1	39,378,617 (32%)	36,527,890 (31%)	2,850,727 (66%)	
T2	41,025,191 (34%)	39,968,463 (34%)	1,056,729 (24%)	
T3	40,972,440 (34%)	40,550,185 (35%)	422,254 (9.8%)	
**DII [mean (SD)]**	1.23 (1.85)	1.21 (1.85)	1.73 (1.74)	**<0.001**
**DII, tertile (%)**				**<0.001**
T1	40,460,537 (34%)	39,421,074 (34%)	1,039,463 (24%)	
T2	40,457,672 (33%)	39,049,336 (33%)	1,408,336 (33%)	
T3	40,458,039 (33%)	38,576,128 (33%)	1,881,911 (43%)	

Mean (SD) for continuous variables: the *P* value was calculated by the weighted Students T-test.

Percentages (weighted *N*, %) for categorical variables: the *P* value was calculated by the weighted chi-square test.

LC9, life’s crucial 9; DII, dietary inflammatory index; PIR, poverty income ratio; CRS, cardiorenal syndrome.

### 3.2 The relationship between LC9 and CRS

**Figure 2:**
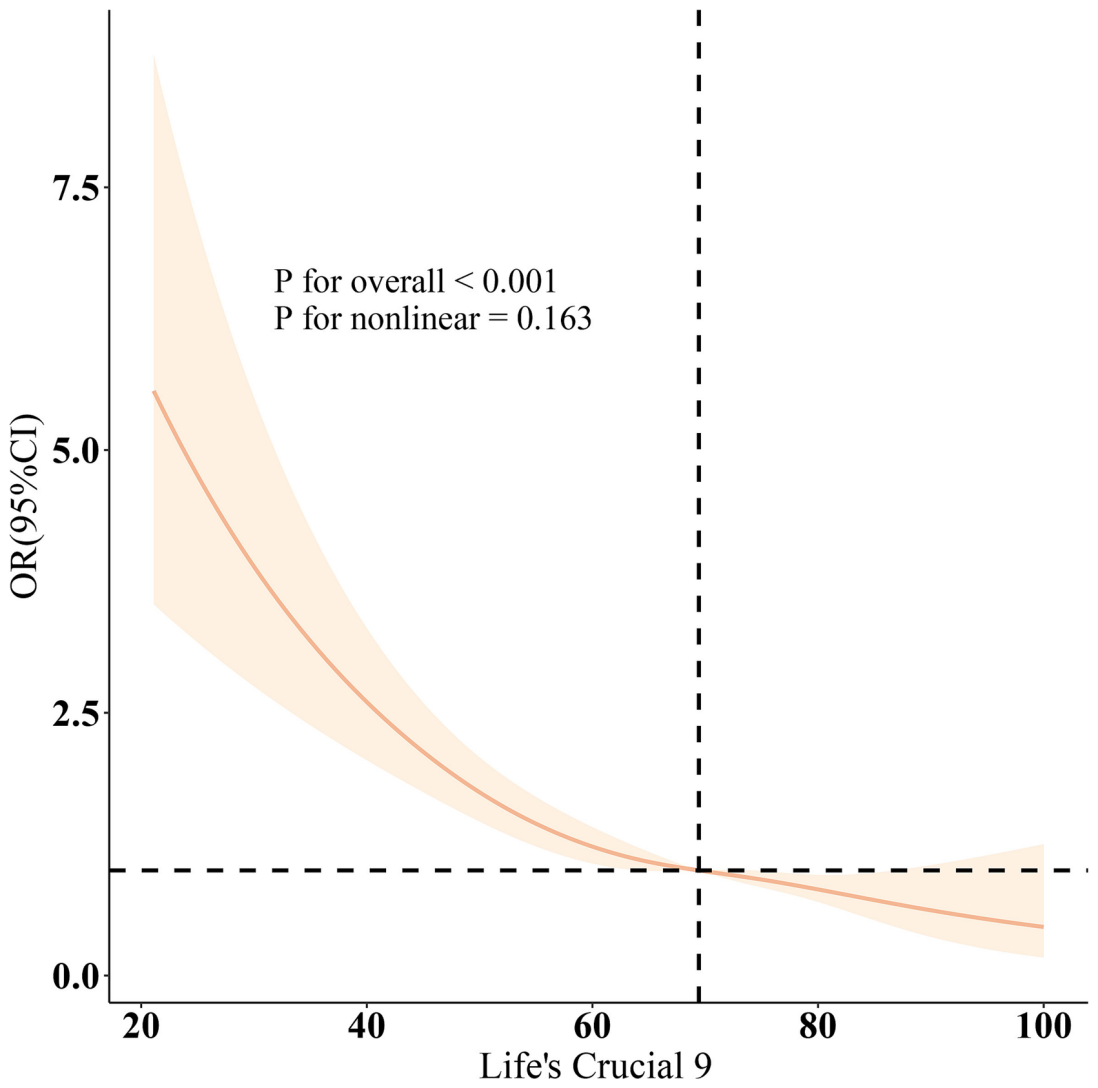
Dose–response relationships between LC9 and CRS. OR (solid lines) and 95% confidence levels (shaded areas) were adjusted for age, gender, education level, marital, PIR, race, smoking, drinking, hypertension, diabetes, and hyperlipidemia.

Table 2 of the multivariable logistic regression model illustrates the correlation between LC9 and CRS. In the modified covariate model 3, weighted logistic regression reveals that a rise in LC9 is negatively correlated with the prevalence of CRS (OR = 0.74, 95% CI = 0.67–0.83, *p* < 0.001). Dividing LC9 into tertiles, with T1 as the reference group, T2 (OR = 0.59, 95% CI = 0.48–0.72, *p* < 0.001) and T3 (OR = 0.57, 95% CI = 0.38–0.88, *p* < 0.001) exhibited a strong negative correlation trend, suggesting that higher LC9 scores are associated with a substantial reduction in the incidence of CRS (*p* < 0.001). In contrast to LC9, Model 3 indicates that when DII is treated as a continuous variable [OR = 1.13, 95% CI = (1.06, 1.20), *p* < 0.001], an increase in DII scores is associated with a higher incidence of CRS. Similarly, dividing DII into tertiles with T1 as the reference group, T2 (OR = 1.25, 95% CI = 1.00–1.55, *p* = 0.046), and T3 (OR = 1.48, 95% CI = 1.15–1.90, *p* = 0.002) showed a significant positive correlation trend. These associations have received consistent support across multiple adjusted models. Figure 2 employs a smooth curve fitting for demonstrating that the relationship of LC9 and CRS is a linear negative correlation (non-linearity = 0.163). The difference between LC9 and DII remained of statistical significance when adjusting variables (*β* = −0.54, 95% CI: −0.57, −0.51, *p* < 0.001) (Table 3), indicating that elevated LC9 scores correlate with diminished DII scores. This study performed multiple imputations to address missing LC9, DII, and CRS data, confirming the stability of the results, which were consistent with the original findings (Supplementary Tables 4, 5).

**Table 2:** Association between LC9, DII, and CRS, NHANES 2005–2018.

Characteristics	Model 1 [OR (95% CI)]	*p-*value	Model 2 [OR (95% CI)]	*p-*value	Model 3 [OR (95% CI)]	*p-*value
**LC9 - CRS**						
Continuous (per 10 scores)	0.56(0.52,0.59)	<0.001	0.61(0.56, 0.66)	<0.001	0.74(0.67, 0.83)	<0.001
Tertile						
T1	1 (ref.)		1 (ref.)		1 (ref.)	
T2	0.34(0.29,0.40)	<0.001	0.41(0.35, 0.49)	<0.001	0.59(0.48, 0.72)	<0.001
T3	0.13(0.09,0.19)	<0.001	0.25(0.17, 0.36)	<0.001	0.57(0.38, 0.88)	<0.001
*P for trend*	<0.001		<0.001		<0.001	
**DII - CRS**						
Continuous	1.18(1.12,1.24)	<0.001	1.17(1.10, 1.24)	<0.001	1.13(1.06, 1.20)	<0.001
Tertile						
T1	1 (ref.)		1 (ref.)		1 (ref.)	
T2	1.37(1.14,1.64)	<0.001	1.38(1.13, 1.69)	0.002	1.25(1.00, 1.55)	0.046
T3	1.85(1.50,2.29)	<0.001	1.74(1.38, 2.19)	<0.001	1.48(1.15, 1.90)	0.002
*P* for trend	<0.001		<0.001		0.002	

Model 1: no covariates were adjusted.

Model 2: age, gender, education level, marital, and race were adjusted.

Model 3: age, gender, education level, marital, PIR, race, smoking, drinking, hypertension, diabetes, and hyperlipidemia were adjusted.

LC9, life’s crucial 9; DII, dietary inflammatory index; PIR, poverty income ratio; CRS, cardiorenal syndrome; OR, odds ratio; CI, confidence interval.

**Table 3:** Multivariate linear regression of LC9 and DII.

	*β*	95%CI	*P*-value
LC9 - DII	−0.54	(−0.57, −0.51)	<0.001

Adjusted for age, gender, education level, marital, PIR, race, smoking, drinking, hypertension, diabetes, and hyperlipidemia.

### 3.3 Subgroup analysis

**Figure 3:**
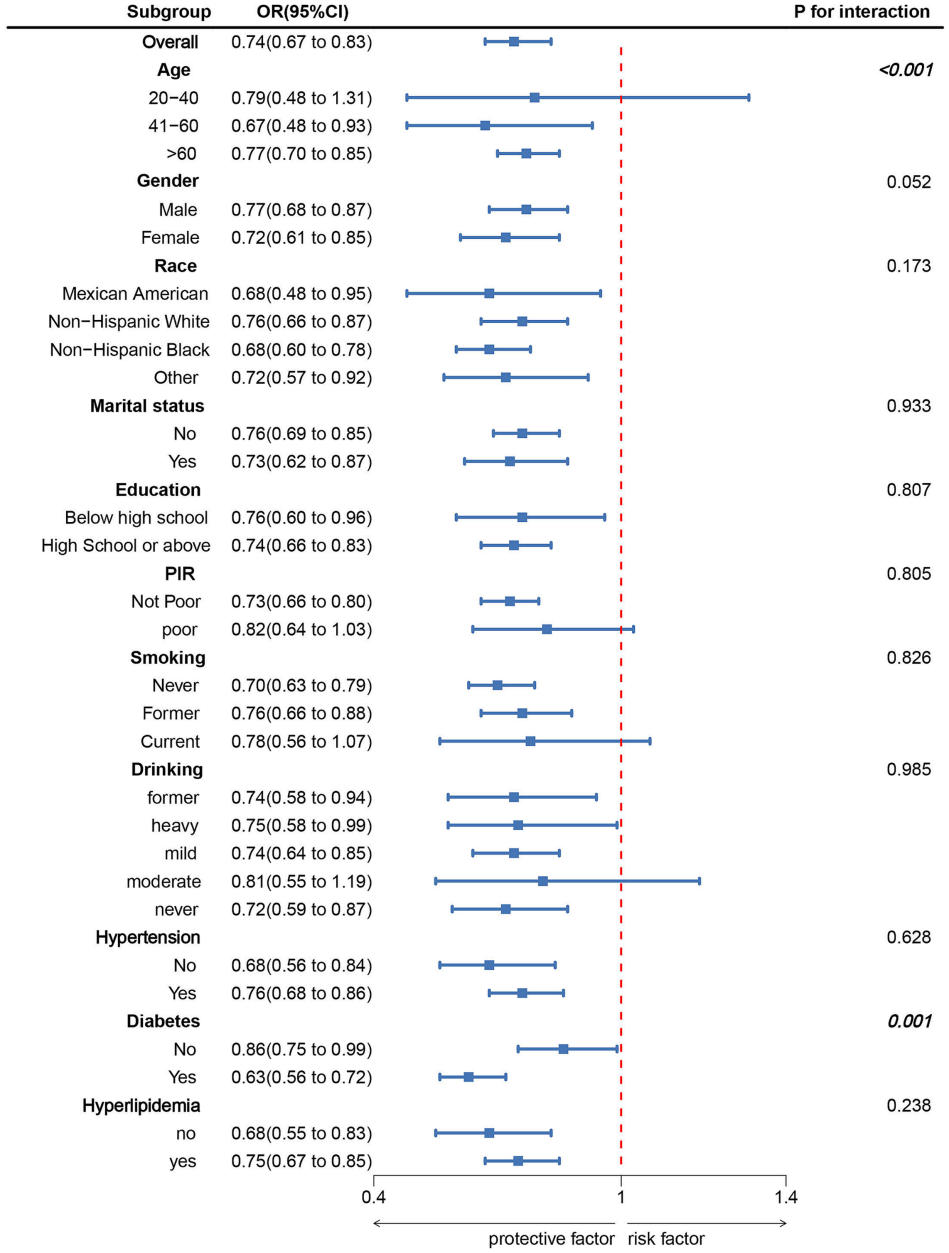
Subgroup analysis between LC9 and CRS. ORs were calculated as per 10-unit increase in LC9. Analyses were adjusted for age, gender, education level, marital, PIR, race, smoking, drinking, hypertension, diabetes, and hyperlipidemia.

Figure 3 displays the subgroup association of LC9 with CRS. The subgroup analysis did not reveal any significant interactions between LC9 and the stratified factors, except for diabetes (*p* > 0.05).

### 3.4 Mediation effect

**Figure 4:**
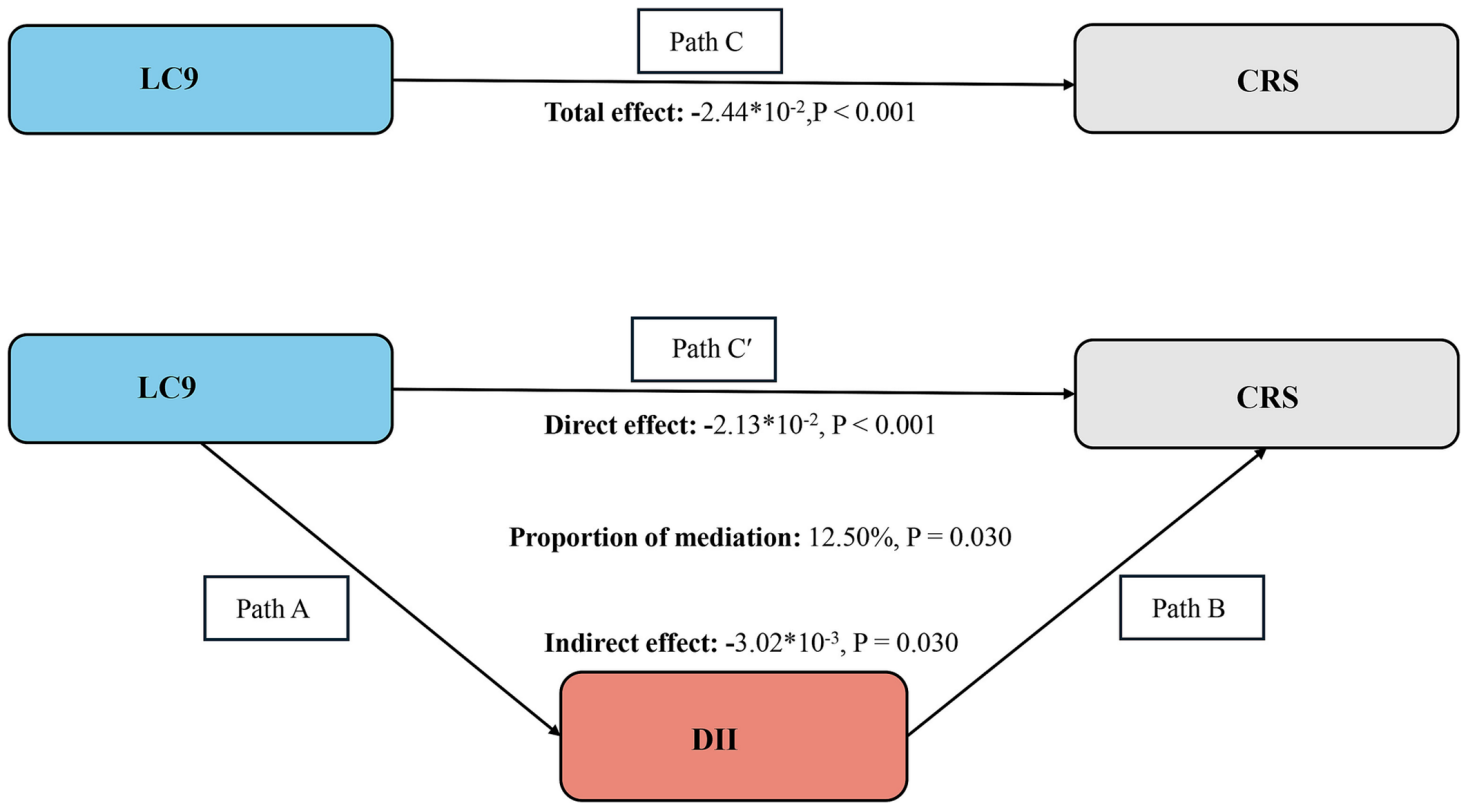
Schematic diagram of the mediation effect analysis. Path C indicates the total effect; path C′ indicates the direct effect. The indirect effect is estimated as the multiplication of paths A and B (path A*B). The mediated proportion is calculated as indirect effect/ (indirect effect + direct effect) × 100%. LC9, life’s crucial 9; DII, dietary inflammatory index; CRS, cardiorenal syndrome. Analyses were adjusted for age, gender, education level, marital, PIR, race, smoking, drinking, hypertension, diabetes, and hyperlipidemia.

The analysis supports the connection between the DII and CRS, and the relationship between LC9 and DII. Our findings meet the prerequisites for conducting a mediation analysis. After adjusting for all covariates, we discovered that DII acts as a mediator. DII (indirect effect = −0.00302, *p* = 0.030; direct effect = −0.0213, *p* < 0.001) accounted for 12.50% of the influence between LC9 and CRS, based on the mediation percentage calculated as (indirect effect / (indirect effect + direct effect) * 100%, *p* = 0.030). Thus, DII is considered a significant mediator in the relationship between LC9 and CRS (Figure 4).

## 4 Discussion

This study, utilizing the NHANES database, identified a negative connection between LC9 and CRS prevalence after controlling for pertinent factors, with DII serving as a mediating factor, thereby corroborating our prediction. The findings from the RCS and subgroup analyses further suggest that elevated LC9 scores are associated with a decrease in the occurrence of CRS. This finding has significant clinical implications for public health interventions and individual health management since it indicates the potential to mitigate CRS risk through the enhancement of lifestyle factors. These findings offer important information for the roles of LC9 and DII in CRS and point to new research directions.

CRS denotes a series of intricate bidirectional pathophysiological mechanisms involving the malfunctioning of the heart and kidneys. The mechanism remains incompletely elucidated, involving numerous complex pathways that concentrate on critical elements such as acute cardiac decompensation, venous congestion, insufficient arterial perfusion, neurohormonal activation, inflammatory response, endothelial dysfunction, and diminished eGFR. The complexities of these pathways encompass high salt affinity, fluid retention, diminished renal clearance, and renal-associated endocrine processes. Furthermore, the difficulties may sustain the pathophysiology (42). The LC9 score is negatively correlated with the risk of CRS, which may be related to the health-promoting effects of the various components of LC9.

LC9 includes nine components: diet, physical activity, tobacco exposure, sleep quality, body mass index, blood pressure, blood glucose, blood cholesterol, and depression, all of which can be adjusted through personal lifestyle choices. While there are currently no studies reporting a direct relationship between LC9 and CRS, the individual components of LC9 have been widely studied and confirmed to be related to CRS or its components, CVD and CKD. First, results from a substantial perspective cohort study with a follow-up period of up to 32 years show that adherence to diverse healthy eating patterns correlates with a diminished risk of CVD (43). A meta-analysis of 18 published studies indicated that each additional point in HEI-2015 reduced the risk CVD mortality by 0.51% (44). A 24-year prospective study in the US showed that participants in the top quintile of the HEI-2015 score had a 17% reduced likelihood of developing of CKD relative to those in the lowest quintile, after adjusting for many covariates (45). Second, it is well-known that a cornerstone of CVD prevention is a healthy lifestyle, including habitual physical exercise (46), with ample scientific evidence supporting that physical exercise is associated with a reduced risk of CVD and CVD mortality (47, 48). Additionally, physical exercise has been repeatedly emphasized as being associated with a reduced prevalence of hypertension and diabetes, which are related to a lower incidence of CKD caused by hypertension and diabetes. Jacob W. Bruinius and colleagues indicated that increased self-reported physical activity correlates with a reduced risk of cardiovascular events and mortality in patients with CKD (49). The findings of a cohort study from the UK suggest that engaging in moderate to vigorous physical activity may significantly reduce the risk of developing CKD and AKI (50). Moreover, smoking is a globally recognized major health threat with significant negative impacts on both CVD and CKD. It damages heart and vascular health by promoting arteriosclerosis, raising blood pressure, accelerating thrombosis, and causing inflammation and oxidative stress, and it also accelerates renal function decline (51–54). Sleep also plays an important role in CRS, with studies confirming that poor quality (both quantity and quality) and sleep disorders correlate with a heightened risk of CVD and CKD (55, 56). Hypertension and diabetes have been confirmed by multiple studies to be associated with CRS (1), and metabolic factors such as body mass index, blood pressure, blood glucose, and blood lipids have been widely studied and confirmed to promote inflammation, oxidative stress, hemodynamic dysfunction, and ischemia, making them risk factors for CVD and CKD (57–61). In CKD patients, every 10-point increase in the LE8 score is associated with a 17% lower risk of all-cause mortality and an 18% lower risk of CVD mortality (17). Finally, from the substantial data of a cross-sectional analysis incorporating over five million people from the China Kadoorie Biobank and around two hundred thousand from the Dongfeng-Tongji cohort, it was found that depression is associated with higher rates of all-cause and CVD mortality compared to individuals without depression. The analysis further supports that depression significantly increases the risk for all-cause and CVD-related deaths even after adjusting for multiple variables (14). A separate investigation revealed a bidirectional association between depression and CVD (62). Research indicates that the inflammation and oxidative nitrosative stress (IO&NS) pathways underlie the shared pathophysiology of CVD and severe depressive disorder. The activation of these pathways may elevate the risk of both diseases and result in shared vulnerability (63). Moreover, depression is prevalent among CKD patients and is strongly linked to the likelihood of negative outcomes (64), with studies confirming a bidirectional relationship between them (65, 66).

This study found that enhancements in LC9 were positively correlated with a decrease in DII, suggesting that a healthy lifestyle correlates with a reduced inflammatory dietary load. Multiple prior investigations have demonstrated that dietary factors substantially influence the global burden of chronic diseases, rendering nutrition a vital modifiable target toward decreasing the incidence of these conditions (67). Dietary habits can affect the likelihood of chronic illnesses through different processes, such as managing gut microbiota, balancing oxidative stress, and maintaining energy balance. The basis of these mechanisms is the potential pro-inflammatory or anti-inflammatory properties of dietary patterns and individual dietary components (68). Studies indicate that elevated DII scores correlate with an increased risk of CVD, and mitigating the pro-inflammatory capacity of the diet may serve as an effective approach for CVD prevention (69). Another study showed that an elevation in DII correlates with a heightened likelihood of CKD stage advancement and a reduction in eGFR. After adjusting for confounding factors, for each unit increase in DII correlates with a 26% rise in the likelihood of CKD stage progression (70). Inflammation is considered a primary factor in the development of CRS. A study using a rat myocardial infarction model to examine the potential mechanisms of renal damage after cardiac dysfunction found that the mRNA expression of inflammatory cytokines IL-6 and TNF-*α* in the kidneys of myocardial infarction rats, as well as microvascular endothelial permeability and renal tubular cell apoptosis, were significantly increased, confirming the important role of the inflammatory response in the occurrence and development of CRS (71). Moreover, as chronic diseases such as hypertension, diabetes, and obesity play a significant role in the management of CRS, there is increasing evidence showing that patients with these chronic conditions have elevated levels of inflammatory biomarkers (including high-sensitivity C-reactive protein, various cytokines, and complement pathway products), which enhance the production of reactive oxygen species (ROS) in cells (72, 73). Therefore, mitigating inflammation through various methods is an effective measure for preventing CRS. Nonetheless, there have been no documented correlations between DII and CRS.

Our study revealed that the DII plays a mediating role between LC9 and CRS, contributing a mediation effect of 12.5%. While this percentage may appear modest, it is important to recognize the complexity of CRS’s pathogenesis, which involves multiple contributing factors. Given this complexity, the fact that dietary improvements are associated with a potential reduction in CRS represents a significant contribution. This underscores the crucial role of dietary habits in regulating systemic inflammation and managing CRS outcomes.

In summary, LC9 is a novel model for assessing cardiovascular health, DII is a dietary index, and CRS is a mechanism-complex disease closely related to a healthy lifestyle. Current research mainly focuses on the relationship between the LC9 model and DII and CRS, a topic that has not been previously explored. This study is the first investigation utilizing the NHANES database to examine the impact of DII on the relationship between LC9 and CRS, offering a novel perspective for forthcoming therapeutic therapies and public health policies. While these findings offer valuable insights into potential lifestyle interventions for CRS prevention, the cross-sectional nature of the study limits causal inferences. Future longitudinal studies are needed to confirm these relationships and explore their underlying mechanisms, providing a foundation for therapeutic and public health strategies.

Furthermore, this study possesses the subsequent limitations: (1) The nature of the cross-sectional data limits causal conclusions. Although LC9 might influence CRS through DII, more comprehensive studies, including prospective cohorts, controlled trials, and animal experiments, are needed to clarify the exact mechanisms involved. (2) Data Collection and Bias: The data for this study were collected primarily through self-administered questionnaires, which can introduce recollection bias. (3) Database Limitations: This study extensively relies on the NHANES database, which is representative of the U.S. population. However, the findings may not completely reflect the circumstances or environmental and genetic diversity found in other countries or regions. This limitation is significant because dietary habits, health behaviors, and disease prevalence can vary widely across different cultural and socio-economic backgrounds, potentially influencing the generalizability of the results to populations outside of the United States. (4) Despite including many covariates based on prior research to strengthen the robustness of our findings, the limitations of cross-sectional studies and the NHANES database prevent us from fully accounting for all potential confounders. Therefore, the results should be interpreted with caution and objectivity.

## Data Availability

The datasets presented in this study can be found in online repositories. The names of the repository/repositories and accession number(s) can be found in the article/Supplementary material.
